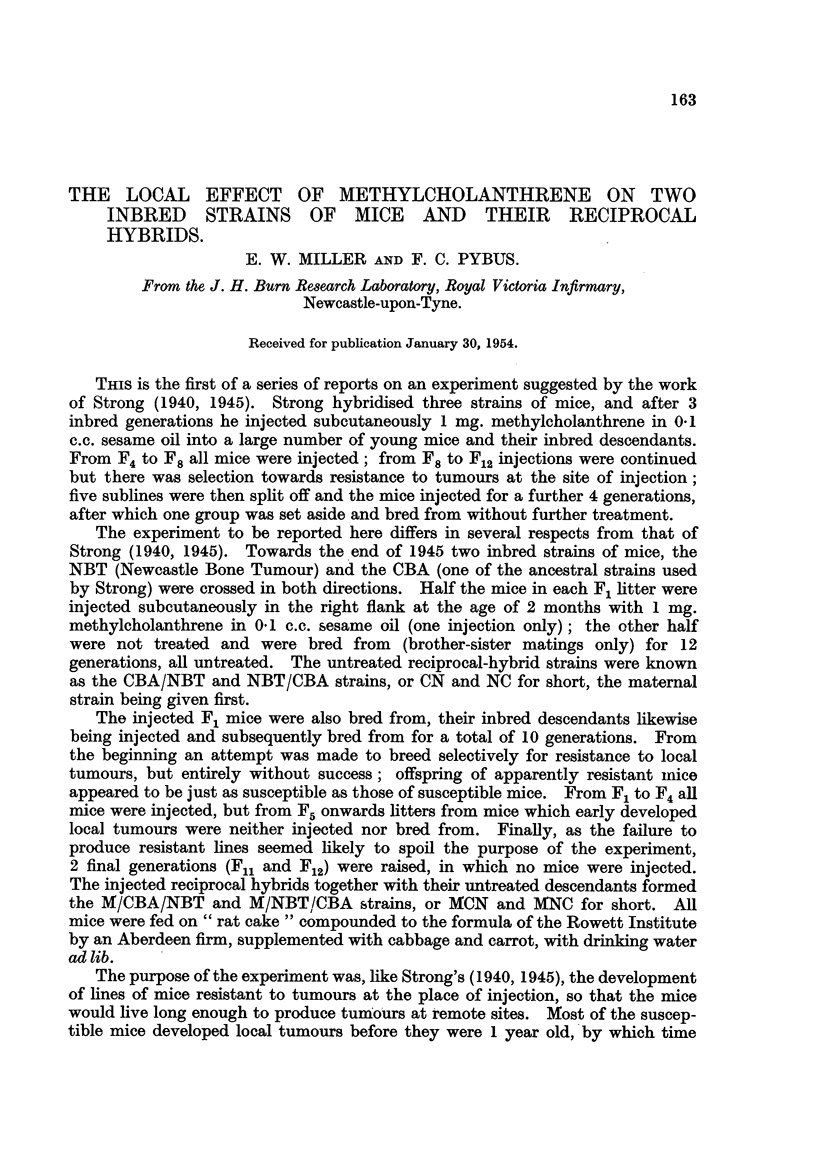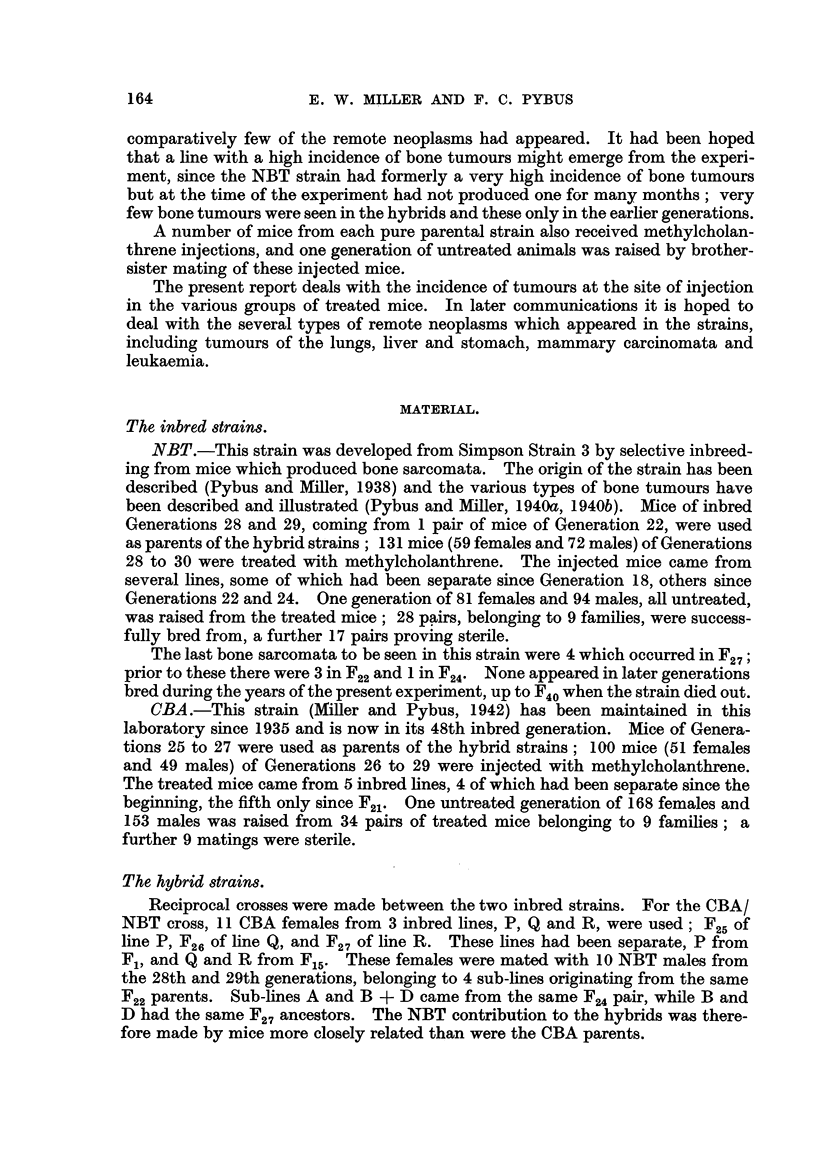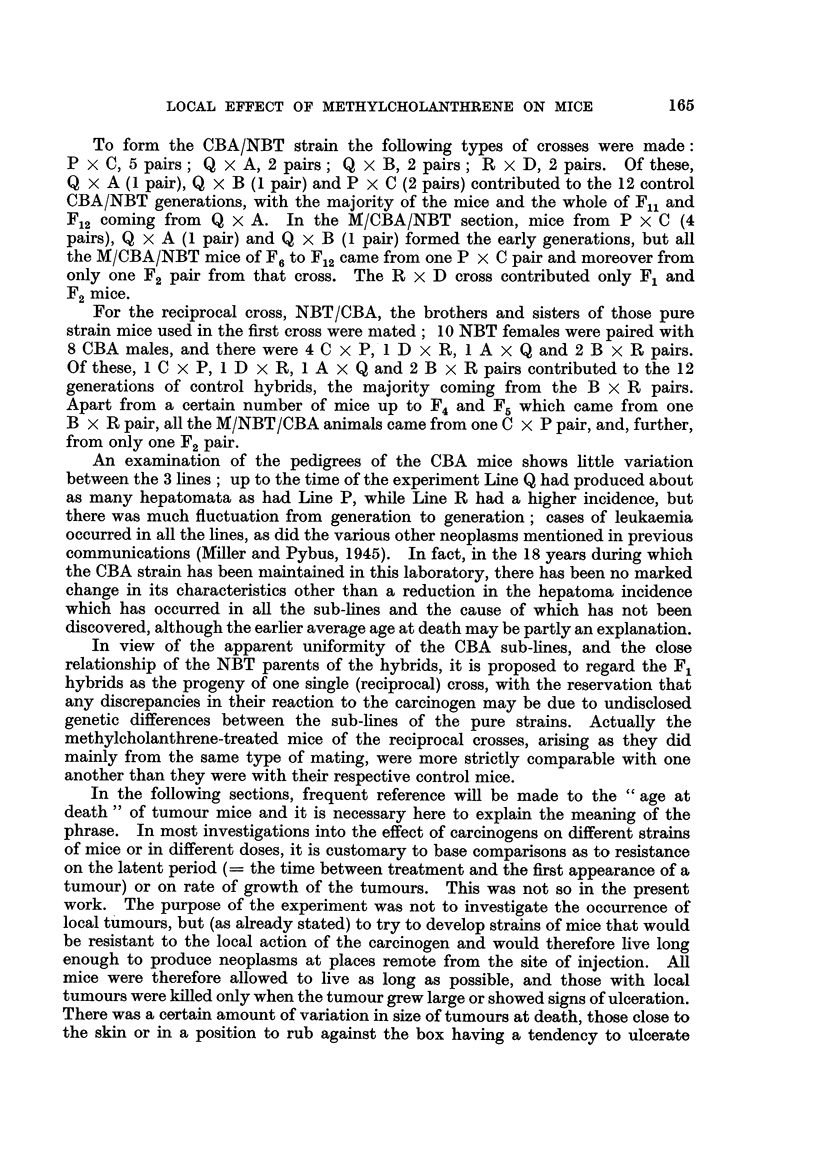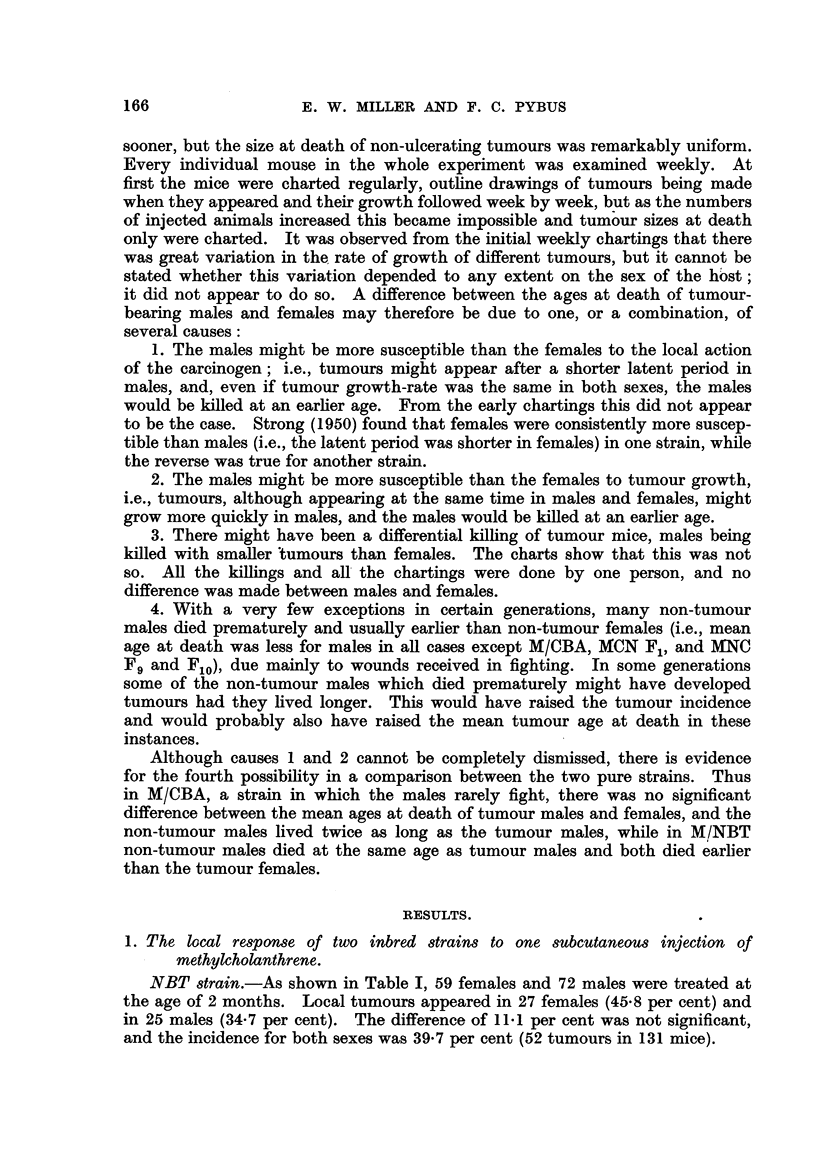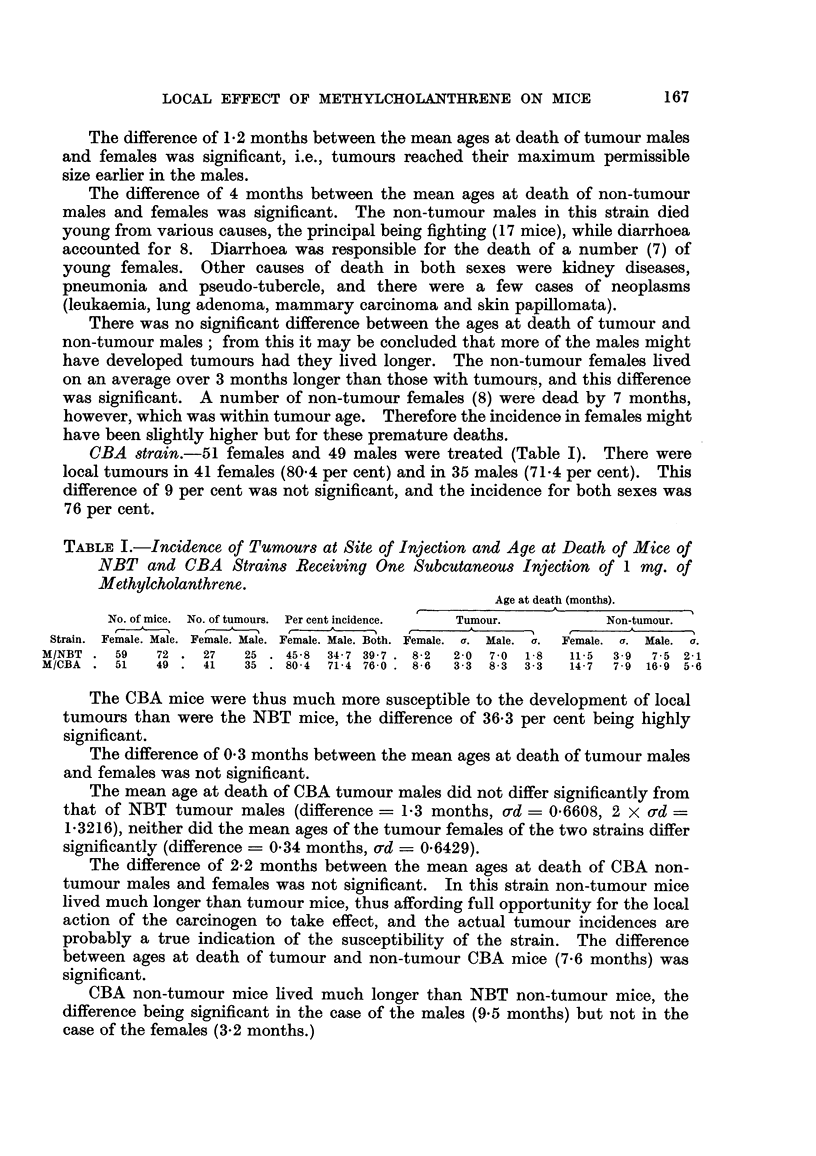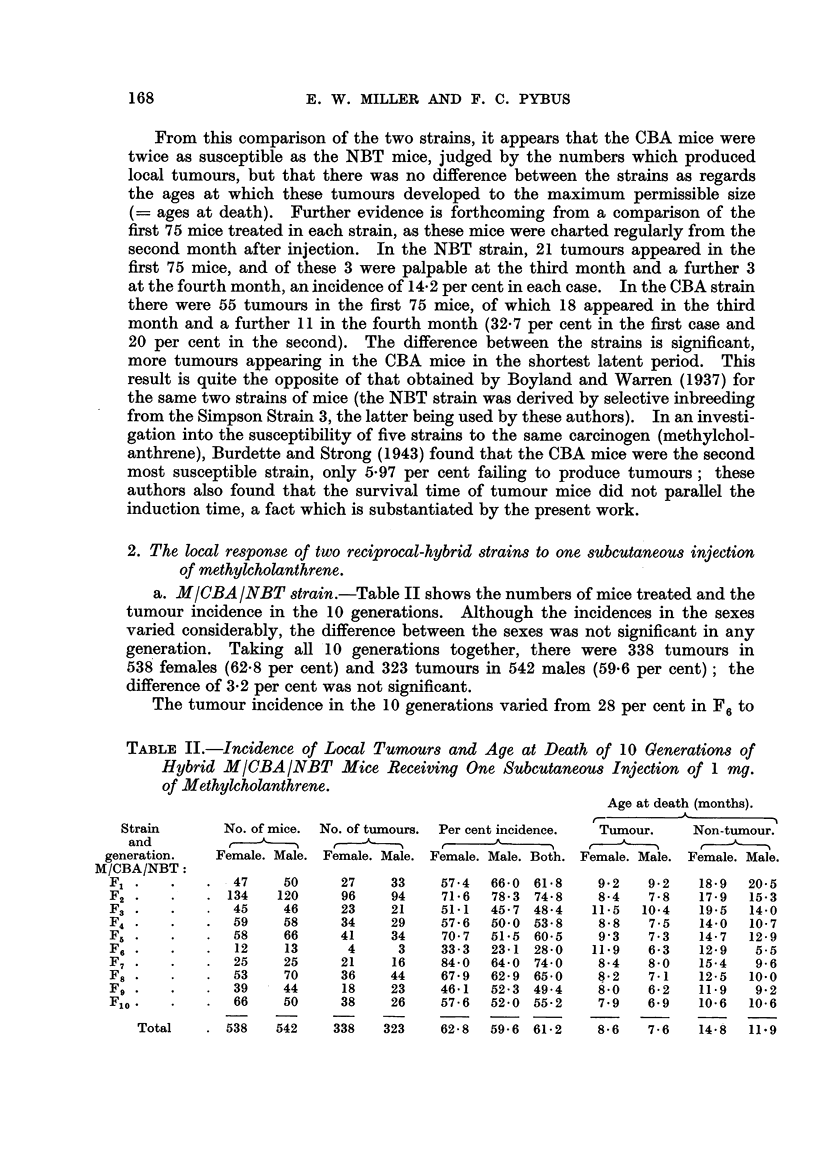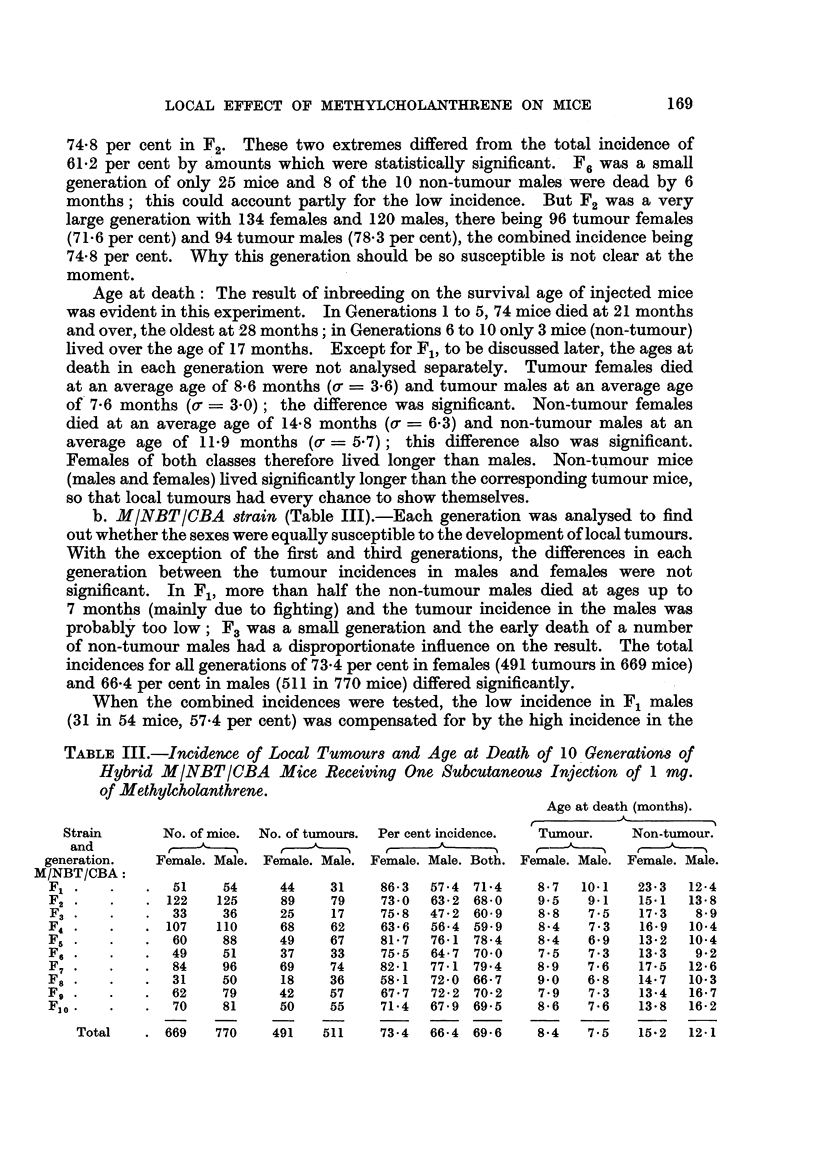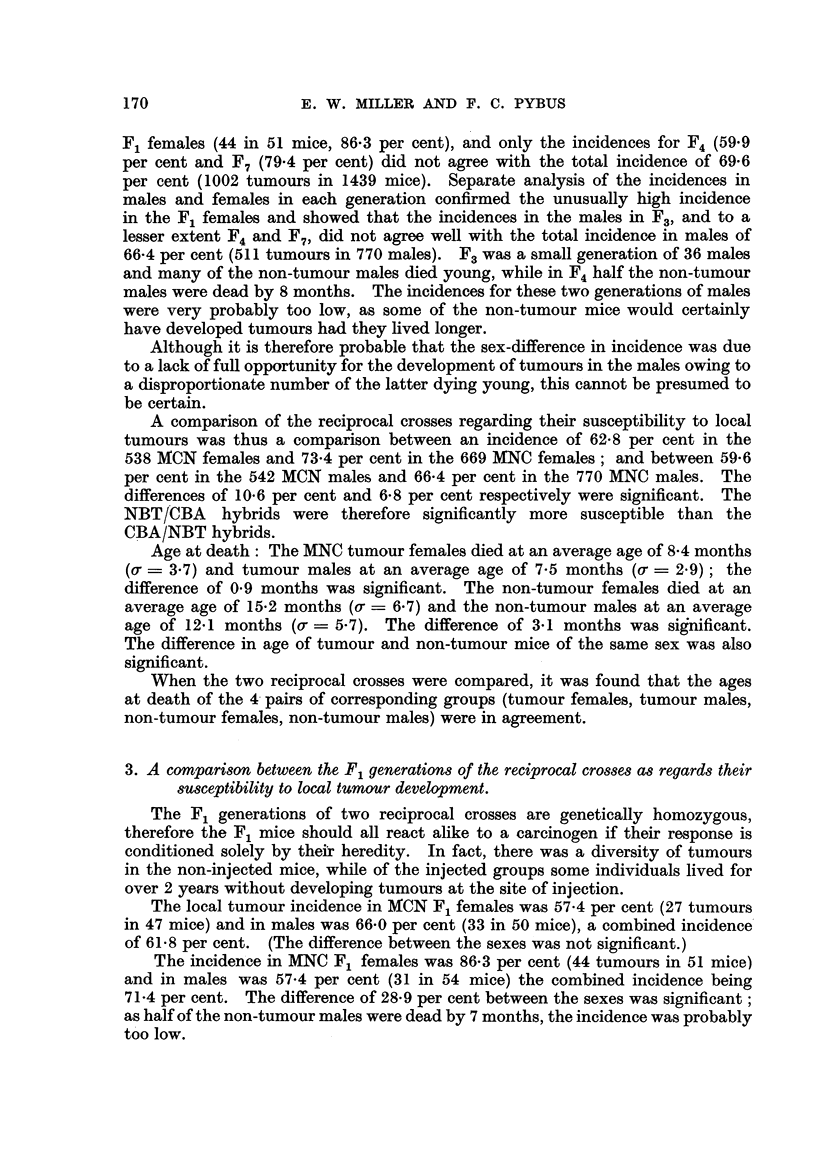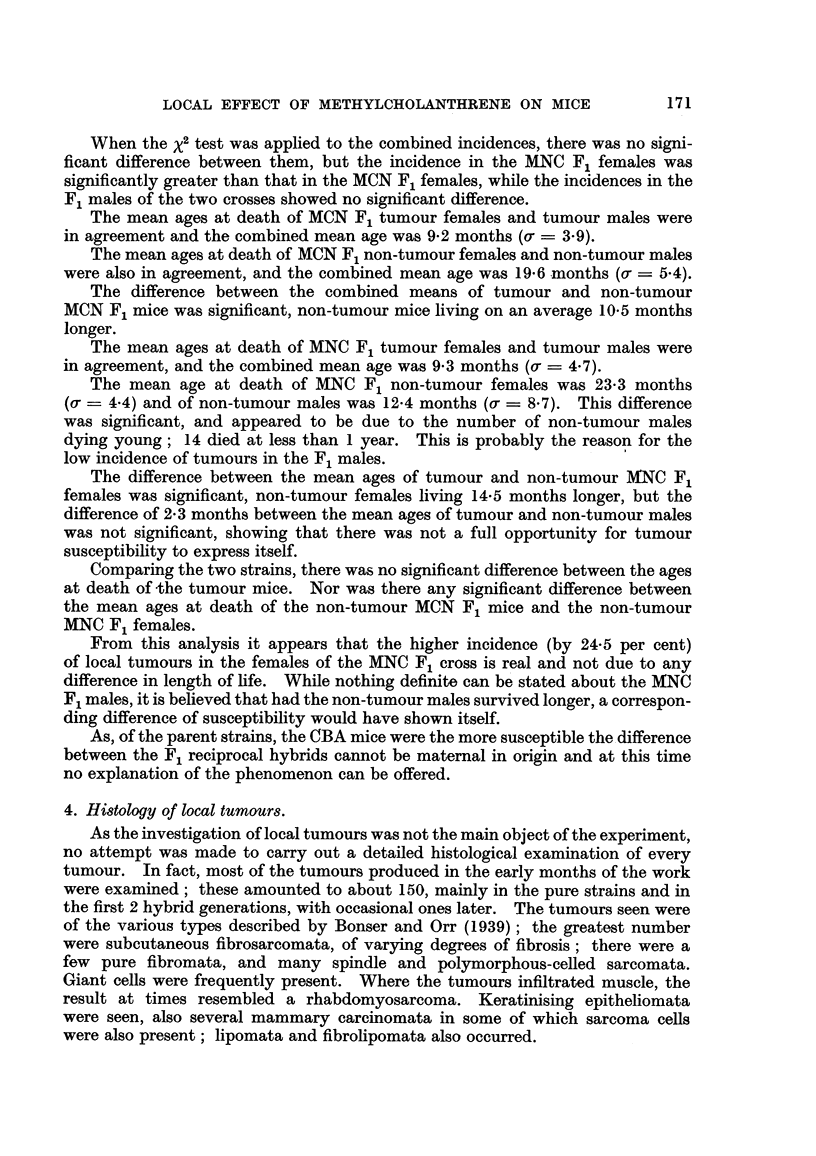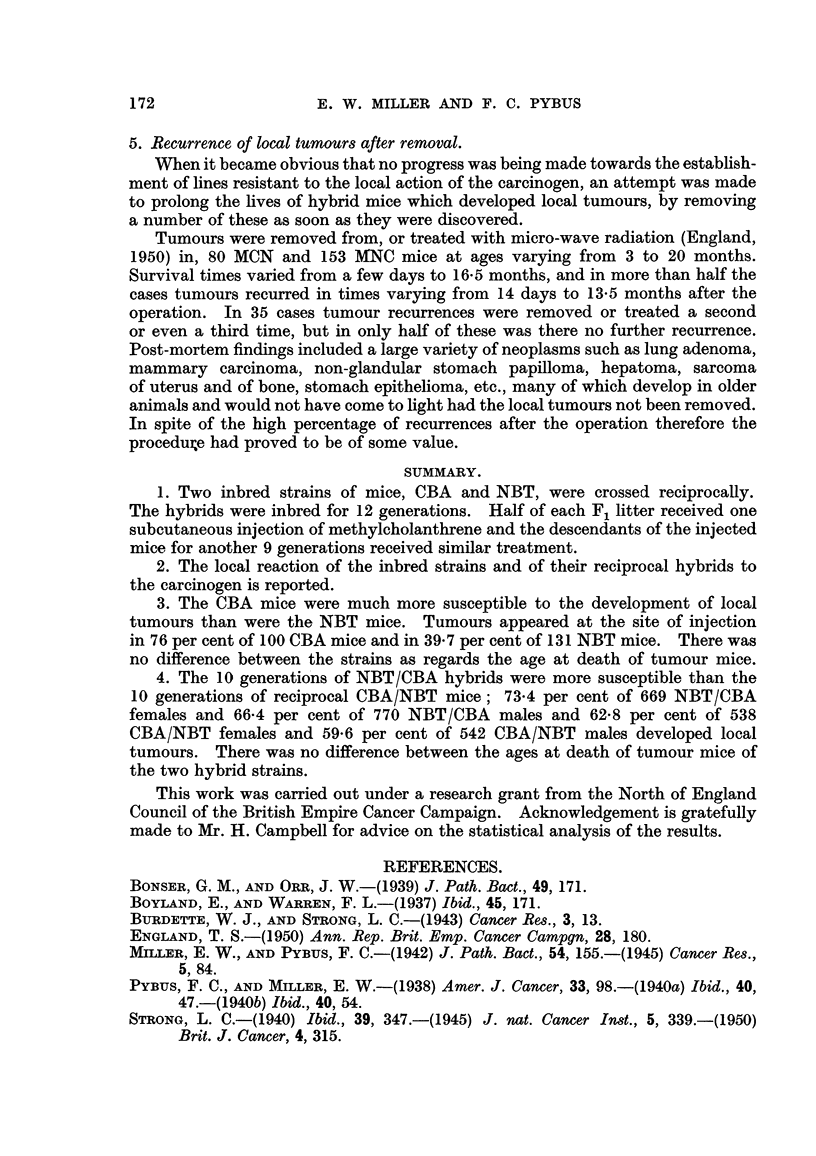# The Local Effect of Methylcholanthrene on two Inbred Strains of Mice and their Reciprocal Hybrids

**DOI:** 10.1038/bjc.1954.15

**Published:** 1954-03

**Authors:** E. W. Miller, F. C. Pybus


### THE LOCAL EFFECT OF METHYLCHOLANTHRENE ON TWO INBRED STRAINS OF MICE AND THEIR RECIPROCAL HYBRIDS.

E. W. MILLER AND F. C. PYBUS.

*From the J. H. Burn Research Laboratory, Royal Victoria Infirmary,* Newcastle-upon-Tyne.

Received for publication January 30, 1954.

THIS is the first of a series of reports on an experiment suggested by the work of Strong (1940, 1945). Strong hybridised three strains of mice, and after 3 inbred generations he injected subcutaneously 1 mg. methylcholanthrene in 0·1 c.c. sesame oil into a large number of young mice and their inbred descendants. From $F_4$ to $F_8$ all mice were injected; from $F_8$ to $F_{12}$ injections were continued but there was selection towards resistance to tumours at the site of injection; five sublines were then split off and the mice injected for a further 4 generations, after which one group was set aside and bred from without further treatment.

The experiment to be reported here differs in several respects from that of Strong (1940, 1945). Towards the end of 1945 two inbred strains of mice, the NBT (Newcastle Bone Tumour) and the CBA (one of the ancestral strains used by Strong) were crossed in both directions. Half the mice in each $F_1$ litter were injected subcutaneously in the right flank at the age of 2 months with 1 mg. methylcholanthrene in 0·1 c.c. sesame oil (one injection only); the other half were not treated and were bred from (brother-sister matings only) for 12 generations, all untreated. The untreated reciprocal-hybrid strains were known as the CBA/NBT and NBT/CBA strains, or CN and NC for short, the maternal strain being given first.

The injected $F_1$ mice were also bred from, their inbred descendants likewise being injected and subsequently bred from for a total of 10 generations. From the beginning an attempt was made to breed selectively for resistance to local tumours, but entirely without success; offspring of apparently resistant mice appeared to be just as susceptible as those of susceptible mice. From $F_1$ to $F_4$ all mice were injected, but from $F_5$ onwards litters from mice which early developed local tumours were neither injected nor bred from. Finally, as the failure to produce resistant lines seemed likely to spoil the purpose of the experiment, 2 final generations ($F_{11}$ and $F_{12}$) were raised, in which no mice were injected. The injected reciprocal hybrids together with their untreated descendants formed the M/CBA/NBT and M/NBT/CBA strains, or MCN and MNC for short. All mice were fed on " rat cake " compounded to the formula of the Rowett Institute by an Aberdeen firm, supplemented with cabbage and carrot, with drinking water *ad lib.*

The purpose of the experiment was, like Strong's (1940, 1945), the development of lines of mice resistant to tumours at the place of injection, so that the mice would live long enough to produce tumours at remote sites. Most of the susceptible mice developed local tumours before they were 1 year old, by which time comparatively few of the remote neoplasms had appeared. It had been hoped that a line with a high incidence of bone tumours might emerge from the experiment, since the NBT strain had formerly a very high incidence of bone tumours but at the time of the experiment had not produced one for many months ; very few bone tumours were seen in the hybrids and these only in the earlier generations.

A number of mice from each pure parental strain also received methylcholanthrene injections, and one generation of untreated animals was raised by brother-sister mating of these injected mice.

The present report deals with the incidence of tumours at the site of injection in the various groups of treated mice. In later communications it is hoped to deal with the several types of remote neoplasms which appeared in the strains, including tumours of the lungs, liver and stomach, mammary carcinomata and leukaemia.

### MATERIAL.

### *The inbred strains.*

*NBT.*—This strain was developed from Simpson Strain 3 by selective inbreeding from mice which produced bone sarcomata. The origin of the strain has been described (Pybus and Miller, 1938) and the various types of bone tumours have been described and illustrated (Pybus and Miller, 1940*a*, 1940*b*). Mice of inbred Generations 28 and 29, coming from 1 pair of mice of Generation 22, were used as parents of the hybrid strains ; 131 mice (59 females and 72 males) of Generations 28 to 30 were treated with methylcholanthrene. The injected mice came from several lines, some of which had been separate since Generation 18, others since Generations 22 and 24. One generation of 81 females and 94 males, all untreated, was raised from the treated mice ; 28 pairs, belonging to 9 families, were successfully bred from, a further 17 pairs proving sterile.

The last bone sarcomata to be seen in this strain were 4 which occurred in $F_{27}$ ; prior to these there were 3 in $F_{22}$ and 1 in $F_{24}$. None appeared in later generations bred during the years of the present experiment, up to $F_{40}$ when the strain died out.

*CBA.*—This strain (Miller and Pybus, 1942) has been maintained in this laboratory since 1935 and is now in its 48th inbred generation. Mice of Generations 25 to 27 were used as parents of the hybrid strains ; 100 mice (51 females and 49 males) of Generations 26 to 29 were injected with methylcholanthrene. The treated mice came from 5 inbred lines, 4 of which had been separate since the beginning, the fifth only since $F_{21}$. One untreated generation of 168 females and 153 males was raised from 34 pairs of treated mice belonging to 9 families ; a further 9 matings were sterile.

### *The hybrid strains.*

Reciprocal crosses were made between the two inbred strains. For the CBA/NBT cross, 11 CBA females from 3 inbred lines, P, Q and R, were used ; $F_{25}$ of line P, $F_{26}$ of line Q, and $F_{27}$ of line R. These lines had been separate, P from $F_1$, and Q and R from $F_{15}$. These females were mated with 10 NBT males from the 28th and 29th generations, belonging to 4 sub-lines originating from the same $F_{22}$ parents. Sub-lines A and B + D came from the same $F_{24}$ pair, while B and D had the same $F_{27}$ ancestors. The NBT contribution to the hybrids was therefore made by mice more closely related than were the CBA parents.

To form the CBA/NBT strain the following types of crosses were made: P × C, 5 pairs; Q × A, 2 pairs; Q × B, 2 pairs; R × D, 2 pairs. Of these, Q × A (1 pair), Q × B (1 pair) and P × C (2 pairs) contributed to the 12 control CBA/NBT generations, with the majority of the mice and the whole of $F_{11}$ and $F_{12}$ coming from Q × A. In the M/CBA/NBT section, mice from P × C (4 pairs), Q × A (1 pair) and Q × B (1 pair) formed the early generations, but all the M/CBA/NBT mice of $F_6$ to $F_{12}$ came from one P × C pair and moreover from only one $F_2$ pair from that cross. The R × D cross contributed only $F_1$ and $F_2$ mice.

For the reciprocal cross, NBT/CBA, the brothers and sisters of those pure strain mice used in the first cross were mated; 10 NBT females were paired with 8 CBA males, and there were 4 C × P, 1 D × R, 1 A × Q and 2 B × R pairs. Of these, 1 C × P, 1 D × R, 1 A × Q and 2 B × R pairs contributed to the 12 generations of control hybrids, the majority coming from the B × R pairs. Apart from a certain number of mice up to $F_4$ and $F_5$ which came from one B × R pair, all the M/NBT/CBA animals came from one C × P pair, and, further, from only one $F_2$ pair.

An examination of the pedigrees of the CBA mice shows little variation between the 3 lines; up to the time of the experiment Line Q had produced about as many hepatomata as had Line P, while Line R had a higher incidence, but there was much fluctuation from generation to generation; cases of leukaemia occurred in all the lines, as did the various other neoplasms mentioned in previous communications (Miller and Pybus, 1945). In fact, in the 18 years during which the CBA strain has been maintained in this laboratory, there has been no marked change in its characteristics other than a reduction in the hepatoma incidence which has occurred in all the sub-lines and the cause of which has not been discovered, although the earlier average age at death may be partly an explanation.

In view of the apparent uniformity of the CBA sub-lines, and the close relationship of the NBT parents of the hybrids, it is proposed to regard the $F_1$ hybrids as the progeny of one single (reciprocal) cross, with the reservation that any discrepancies in their reaction to the carcinogen may be due to undisclosed genetic differences between the sub-lines of the pure strains. Actually the methylcholanthrene-treated mice of the reciprocal crosses, arising as they did mainly from the same type of mating, were more strictly comparable with one another than they were with their respective control mice.

In the following sections, frequent reference will be made to the "age at death" of tumour mice and it is necessary here to explain the meaning of the phrase. In most investigations into the effect of carcinogens on different strains of mice or in different doses, it is customary to base comparisons as to resistance on the latent period (= the time between treatment and the first appearance of a tumour) or on rate of growth of the tumours. This was not so in the present work. The purpose of the experiment was not to investigate the occurrence of local tumours, but (as already stated) to try to develop strains of mice that would be resistant to the local action of the carcinogen and would therefore live long enough to produce neoplasms at places remote from the site of injection. All mice were therefore allowed to live as long as possible, and those with local tumours were killed only when the tumour grew large or showed signs of ulceration. There was a certain amount of variation in size of tumours at death, those close to the skin or in a position to rub against the box having a tendency to ulcerate sooner, but the size at death of non-ulcerating tumours was remarkably uniform. Every individual mouse in the whole experiment was examined weekly. At first the mice were charted regularly, outline drawings of tumours being made when they appeared and their growth followed week by week, but as the numbers of injected animals increased this became impossible and tumour sizes at death only were charted. It was observed from the initial weekly chartings that there was great variation in the rate of growth of different tumours, but it cannot be stated whether this variation depended to any extent on the sex of the host; it did not appear to do so. A difference between the ages at death of tumour-bearing males and females may therefore be due to one, or a combination, of several causes:

1. The males might be more susceptible than the females to the local action of the carcinogen; i.e., tumours might appear after a shorter latent period in males, and, even if tumour growth-rate was the same in both sexes, the males would be killed at an earlier age. From the early chartings this did not appear to be the case. Strong (1950) found that females were consistently more susceptible than males (i.e., the latent period was shorter in females) in one strain, while the reverse was true for another strain.

2. The males might be more susceptible than the females to tumour growth, i.e., tumours, although appearing at the same time in males and females, might grow more quickly in males, and the males would be killed at an earlier age.

3. There might have been a differential killing of tumour mice, males being killed with smaller tumours than females. The charts show that this was not so. All the killings and all the chartings were done by one person, and no difference was made between males and females.

4. With a very few exceptions in certain generations, many non-tumour males died prematurely and usually earlier than non-tumour females (i.e., mean age at death was less for males in all cases except M/CBA, MCN $F_1$, and MNC $F_9$ and $F_{10}$), due mainly to wounds received in fighting. In some generations some of the non-tumour males which died prematurely might have developed tumours had they lived longer. This would have raised the tumour incidence and would probably also have raised the mean tumour age at death in these instances.

Although causes 1 and 2 cannot be completely dismissed, there is evidence for the fourth possibility in a comparison between the two pure strains. Thus in M/CBA, a strain in which the males rarely fight, there was no significant difference between the mean ages at death of tumour males and females, and the non-tumour males lived twice as long as the tumour males, while in M/NBT non-tumour males died at the same age as tumour males and both died earlier than the tumour females.

### RESULTS.

### 1. *The local response of two inbred strains to one subcutaneous injection of methylcholanthrene.*

*NBT strain.*—As shown in Table I, 59 females and 72 males were treated at the age of 2 months. Local tumours appeared in 27 females (45·8 per cent) and in 25 males (34·7 per cent). The difference of 11·1 per cent was not significant, and the incidence for both sexes was 39·7 per cent (52 tumours in 131 mice).

The difference of 1·2 months between the mean ages at death of tumour males and females was significant, i.e., tumours reached their maximum permissible size earlier in the males.

The difference of 4 months between the mean ages at death of non-tumour males and females was significant. The non-tumour males in this strain died young from various causes, the principal being fighting (17 mice), while diarrhoea accounted for 8. Diarrhoea was responsible for the death of a number (7) of young females. Other causes of death in both sexes were kidney diseases, pneumonia and pseudo-tubercle, and there were a few cases of neoplasms (leukaemia, lung adenoma, mammary carcinoma and skin papillomata).

There was no significant difference between the ages at death of tumour and non-tumour males ; from this it may be concluded that more of the males might have developed tumours had they lived longer. The non-tumour females lived on an average over 3 months longer than those with tumours, and this difference was significant. A number of non-tumour females (8) were dead by 7 months, however, which was within tumour age. Therefore the incidence in females might have been slightly higher but for these premature deaths.

*CBA strain.*—51 females and 49 males were treated (Table I). There were local tumours in 41 females (80·4 per cent) and in 35 males (71·4 per cent). This difference of 9 per cent was not significant, and the incidence for both sexes was 76 per cent.

TABLE I.—*Incidence of Tumours at Site of Injection and Age at Death of Mice of NBT and CBA Strains Receiving One Subcutaneous Injection of* 1 *mg. of Methylcholanthrene.*

| | | | | | | | | Age at death (months). | | | | | | | |
|---|---|---|---|---|---|---|---|---|---|---|---|---|---|---|---|
| | No. of mice. | | No. of tumours. | | Per cent incidence. | | | Tumour. | | | | Non-tumour. | | | |
| Strain. | Female. | Male. | Female. | Male. | Female. | Male. | Both. | Female. | $\sigma$. | Male. | $\sigma$. | Female. | $\sigma$. | Male. | $\sigma$. |
| M/NBT | 59 | 72 | 27 | 25 | 45·8 | 34·7 | 39·7 | 8·2 | 2·0 | 7·0 | 1·8 | 11·5 | 3·9 | 7·5 | 2·1 |
| M/CBA | 51 | 49 | 41 | 35 | 80·4 | 71·4 | 76·0 | 8·6 | 3·3 | 8·3 | 3·3 | 14·7 | 7·9 | 16·9 | 5·6 |

The CBA mice were thus much more susceptible to the development of local tumours than were the NBT mice, the difference of 36·3 per cent being highly significant.

The difference of 0·3 months between the mean ages at death of tumour males and females was not significant.

The mean age at death of CBA tumour males did not differ significantly from that of NBT tumour males (difference = 1·3 months, $\sigma d = 0{\cdot}6608$, $2 \times \sigma d = 1{\cdot}3216$), neither did the mean ages of the tumour females of the two strains differ significantly (difference = 0·34 months, $\sigma d = 0{\cdot}6429$).

The difference of 2·2 months between the mean ages at death of CBA non-tumour males and females was not significant. In this strain non-tumour mice lived much longer than tumour mice, thus affording full opportunity for the local action of the carcinogen to take effect, and the actual tumour incidences are probably a true indication of the susceptibility of the strain. The difference between ages at death of tumour and non-tumour CBA mice (7·6 months) was significant.

CBA non-tumour mice lived much longer than NBT non-tumour mice, the difference being significant in the case of the males (9·5 months) but not in the case of the females (3·2 months.)

From this comparison of the two strains, it appears that the CBA mice were twice as susceptible as the NBT mice, judged by the numbers which produced local tumours, but that there was no difference between the strains as regards the ages at which these tumours developed to the maximum permissible size (= ages at death). Further evidence is forthcoming from a comparison of the first 75 mice treated in each strain, as these mice were charted regularly from the second month after injection. In the NBT strain, 21 tumours appeared in the first 75 mice, and of these 3 were palpable at the third month and a further 3 at the fourth month, an incidence of 14·2 per cent in each case. In the CBA strain there were 55 tumours in the first 75 mice, of which 18 appeared in the third month and a further 11 in the fourth month (32·7 per cent in the first case and 20 per cent in the second). The difference between the strains is significant, more tumours appearing in the CBA mice in the shortest latent period. This result is quite the opposite of that obtained by Boyland and Warren (1937) for the same two strains of mice (the NBT strain was derived by selective inbreeding from the Simpson Strain 3, the latter being used by these authors). In an investigation into the susceptibility of five strains to the same carcinogen (methylcholanthrene), Burdette and Strong (1943) found that the CBA mice were the second most susceptible strain, only 5·97 per cent failing to produce tumours ; these authors also found that the survival time of tumour mice did not parallel the induction time, a fact which is substantiated by the present work.

2. *The local response of two reciprocal-hybrid strains to one subcutaneous injection of methylcholanthrene.*

a. *M/CBA/NBT strain.*—Table II shows the numbers of mice treated and the tumour incidence in the 10 generations. Although the incidences in the sexes varied considerably, the difference between the sexes was not significant in any generation. Taking all 10 generations together, there were 338 tumours in 538 females (62·8 per cent) and 323 tumours in 542 males (59·6 per cent) ; the difference of 3·2 per cent was not significant.

The tumour incidence in the 10 generations varied from 28 per cent in $F_6$ to

TABLE II.—*Incidence of Local Tumours and Age at Death of* 10 *Generations of Hybrid M/CBA/NBT Mice Receiving One Subcutaneous Injection of* 1 *mg. of Methylcholanthrene.*

| Strain and generation. | No. of mice. | | No. of tumours. | | Per cent incidence. | | | Age at death (months). Tumour. | | Age at death (months). Non-tumour. | |
|---|---|---|---|---|---|---|---|---|---|---|---|
| | Female. | Male. | Female. | Male. | Female. | Male. | Both. | Female. | Male. | Female. | Male. |
| M/CBA/NBT : | | | | | | | | | | | |
| $F_1$ . . . | 47 | 50 | 27 | 33 | 57·4 | 66·0 | 61·8 | 9·2 | 9·2 | 18·9 | 20·5 |
| $F_2$ . . . | 134 | 120 | 96 | 94 | 71·6 | 78·3 | 74·8 | 8·4 | 7·8 | 17·9 | 15·3 |
| $F_3$ . . . | 45 | 46 | 23 | 21 | 51·1 | 45·7 | 48·4 | 11·5 | 10·4 | 19·5 | 14·0 |
| $F_4$ . . . | 59 | 58 | 34 | 29 | 57·6 | 50·0 | 53·8 | 8·8 | 7·5 | 14·0 | 10·7 |
| $F_5$ . . . | 58 | 66 | 41 | 34 | 70·7 | 51·5 | 60·5 | 9·3 | 7·3 | 14·7 | 12·9 |
| $F_6$ . . . | 12 | 13 | 4 | 3 | 33·3 | 23·1 | 28·0 | 11·9 | 6·3 | 12·9 | 5·5 |
| $F_7$ . . . | 25 | 25 | 21 | 16 | 84·0 | 64·0 | 74·0 | 8·4 | 8·0 | 15·4 | 9·6 |
| $F_8$ . . . | 53 | 70 | 36 | 44 | 67·9 | 62·9 | 65·0 | 8·2 | 7·1 | 12·5 | 10·0 |
| $F_9$ . . . | 39 | 44 | 18 | 23 | 46·1 | 52·3 | 49·4 | 8·0 | 6·2 | 11·9 | 9·2 |
| $F_{10}$ . . . | 66 | 50 | 38 | 26 | 57·6 | 52·0 | 55·2 | 7·9 | 6·9 | 10·6 | 10·6 |
| Total . | 538 | 542 | 338 | 323 | 62·8 | 59·6 | 61·2 | 8·6 | 7·6 | 14·8 | 11·9 |

74·8 per cent in $F_2$. These two extremes differed from the total incidence of 61·2 per cent by amounts which were statistically significant. $F_6$ was a small generation of only 25 mice and 8 of the 10 non-tumour males were dead by 6 months; this could account partly for the low incidence. But $F_2$ was a very large generation with 134 females and 120 males, there being 96 tumour females (71·6 per cent) and 94 tumour males (78·3 per cent), the combined incidence being 74·8 per cent. Why this generation should be so susceptible is not clear at the moment.

Age at death: The result of inbreeding on the survival age of injected mice was evident in this experiment. In Generations 1 to 5, 74 mice died at 21 months and over, the oldest at 28 months; in Generations 6 to 10 only 3 mice (non-tumour) lived over the age of 17 months. Except for $F_1$, to be discussed later, the ages at death in each generation were not analysed separately. Tumour females died at an average age of 8·6 months ($\sigma = 3{\cdot}6$) and tumour males at an average age of 7·6 months ($\sigma = 3{\cdot}0$); the difference was significant. Non-tumour females died at an average age of 14·8 months ($\sigma = 6{\cdot}3$) and non-tumour males at an average age of 11·9 months ($\sigma = 5{\cdot}7$); this difference also was significant. Females of both classes therefore lived longer than males. Non-tumour mice (males and females) lived significantly longer than the corresponding tumour mice, so that local tumours had every chance to show themselves.

b. *M/NBT/CBA strain* (Table III).—Each generation was analysed to find out whether the sexes were equally susceptible to the development of local tumours. With the exception of the first and third generations, the differences in each generation between the tumour incidences in males and females were not significant. In $F_1$, more than half the non-tumour males died at ages up to 7 months (mainly due to fighting) and the tumour incidence in the males was probably too low; $F_3$ was a small generation and the early death of a number of non-tumour males had a disproportionate influence on the result. The total incidences for all generations of 73·4 per cent in females (491 tumours in 669 mice) and 66·4 per cent in males (511 in 770 mice) differed significantly.

When the combined incidences were tested, the low incidence in $F_1$ males (31 in 54 mice, 57·4 per cent) was compensated for by the high incidence in the

TABLE III.—*Incidence of Local Tumours and Age at Death of* 10 *Generations of Hybrid M/NBT/CBA Mice Receiving One Subcutaneous Injection of* 1 *mg. of Methylcholanthrene.*

| Strain and generation. | No. of mice. | | No. of tumours. | | Per cent incidence. | | | Age at death (months). Tumour. | | Age at death (months). Non-tumour. | |
|---|---|---|---|---|---|---|---|---|---|---|---|
| | Female. | Male. | Female. | Male. | Female. | Male. | Both. | Female. | Male. | Female. | Male. |
| M/NBT/CBA: | | | | | | | | | | | |
| $F_1$ . . . | 51 | 54 | 44 | 31 | 86·3 | 57·4 | 71·4 | 8·7 | 10·1 | 23·3 | 12·4 |
| $F_2$ . . . | 122 | 125 | 89 | 79 | 73·0 | 63·2 | 68·0 | 9·5 | 9·1 | 15·1 | 13·8 |
| $F_3$ . . . | 33 | 36 | 25 | 17 | 75·8 | 47·2 | 60·9 | 8·8 | 7·5 | 17·3 | 8·9 |
| $F_4$ . . . | 107 | 110 | 68 | 62 | 63·6 | 56·4 | 59·9 | 8·4 | 7·3 | 16·9 | 10·4 |
| $F_5$ . . . | 60 | 88 | 49 | 67 | 81·7 | 76·1 | 78·4 | 8·4 | 6·9 | 13·2 | 10·4 |
| $F_6$ . . . | 49 | 51 | 37 | 33 | 75·5 | 64·7 | 70·0 | 7·5 | 7·3 | 13·3 | 9·2 |
| $F_7$ . . . | 84 | 96 | 69 | 74 | 82·1 | 77·1 | 79·4 | 8·9 | 7·6 | 17·5 | 12·6 |
| $F_8$ . . . | 31 | 50 | 18 | 36 | 58·1 | 72·0 | 66·7 | 9·0 | 6·8 | 14·7 | 10·3 |
| $F_9$ . . . | 62 | 79 | 42 | 57 | 67·7 | 72·2 | 70·2 | 7·9 | 7·3 | 13·4 | 16·7 |
| $F_{10}$ . . . | 70 | 81 | 50 | 55 | 71·4 | 67·9 | 69·5 | 8·6 | 7·6 | 13·8 | 16·2 |
| Total . | 669 | 770 | 491 | 511 | 73·4 | 66·4 | 69·6 | 8·4 | 7·5 | 15·2 | 12·1 |

$F_1$ females (44 in 51 mice, 86·3 per cent), and only the incidences for $F_4$ (59·9 per cent and $F_7$ (79·4 per cent) did not agree with the total incidence of 69·6 per cent (1002 tumours in 1439 mice). Separate analysis of the incidences in males and females in each generation confirmed the unusually high incidence in the $F_1$ females and showed that the incidences in the males in $F_3$, and to a lesser extent $F_4$ and $F_7$, did not agree well with the total incidence in males of 66·4 per cent (511 tumours in 770 males). $F_3$ was a small generation of 36 males and many of the non-tumour males died young, while in $F_4$ half the non-tumour males were dead by 8 months. The incidences for these two generations of males were very probably too low, as some of the non-tumour mice would certainly have developed tumours had they lived longer.

Although it is therefore probable that the sex-difference in incidence was due to a lack of full opportunity for the development of tumours in the males owing to a disproportionate number of the latter dying young, this cannot be presumed to be certain.

A comparison of the reciprocal crosses regarding their susceptibility to local tumours was thus a comparison between an incidence of 62·8 per cent in the 538 MCN females and 73·4 per cent in the 669 MNC females ; and between 59·6 per cent in the 542 MCN males and 66·4 per cent in the 770 MNC males. The differences of 10·6 per cent and 6·8 per cent respectively were significant. The NBT/CBA hybrids were therefore significantly more susceptible than the CBA/NBT hybrids.

Age at death : The MNC tumour females died at an average age of 8·4 months ($\sigma = 3·7$) and tumour males at an average age of 7·5 months ($\sigma = 2·9$) ; the difference of 0·9 months was significant. The non-tumour females died at an average age of 15·2 months ($\sigma = 6·7$) and the non-tumour males at an average age of 12·1 months ($\sigma = 5·7$). The difference of 3·1 months was significant. The difference in age of tumour and non-tumour mice of the same sex was also significant.

When the two reciprocal crosses were compared, it was found that the ages at death of the 4 pairs of corresponding groups (tumour females, tumour males, non-tumour females, non-tumour males) were in agreement.

3. *A comparison between the $F_1$ generations of the reciprocal crosses as regards their susceptibility to local tumour development.*

The $F_1$ generations of two reciprocal crosses are genetically homozygous, therefore the $F_1$ mice should all react alike to a carcinogen if their response is conditioned solely by their heredity. In fact, there was a diversity of tumours in the non-injected mice, while of the injected groups some individuals lived for over 2 years without developing tumours at the site of injection.

The local tumour incidence in MCN $F_1$ females was 57·4 per cent (27 tumours in 47 mice) and in males was 66·0 per cent (33 in 50 mice), a combined incidence of 61·8 per cent. (The difference between the sexes was not significant.)

The incidence in MNC $F_1$ females was 86·3 per cent (44 tumours in 51 mice) and in males was 57·4 per cent (31 in 54 mice) the combined incidence being 71·4 per cent. The difference of 28·9 per cent between the sexes was significant ; as half of the non-tumour males were dead by 7 months, the incidence was probably too low.

When the $\chi^2$ test was applied to the combined incidences, there was no significant difference between them, but the incidence in the MNC $F_1$ females was significantly greater than that in the MCN $F_1$ females, while the incidences in the $F_1$ males of the two crosses showed no significant difference.

The mean ages at death of MCN $F_1$ tumour females and tumour males were in agreement and the combined mean age was 9·2 months ($\sigma = 3·9$).

The mean ages at death of MCN $F_1$ non-tumour females and non-tumour males were also in agreement, and the combined mean age was 19·6 months ($\sigma = 5·4$).

The difference between the combined means of tumour and non-tumour MCN $F_1$ mice was significant, non-tumour mice living on an average 10·5 months longer.

The mean ages at death of MNC $F_1$ tumour females and tumour males were in agreement, and the combined mean age was 9·3 months ($\sigma = 4·7$).

The mean age at death of MNC $F_1$ non-tumour females was 23·3 months ($\sigma = 4·4$) and of non-tumour males was 12·4 months ($\sigma = 8·7$). This difference was significant, and appeared to be due to the number of non-tumour males dying young ; 14 died at less than 1 year. This is probably the reason for the low incidence of tumours in the $F_1$ males.

The difference between the mean ages of tumour and non-tumour MNC $F_1$ females was significant, non-tumour females living 14·5 months longer, but the difference of 2·3 months between the mean ages of tumour and non-tumour males was not significant, showing that there was not a full opportunity for tumour susceptibility to express itself.

Comparing the two strains, there was no significant difference between the ages at death of the tumour mice. Nor was there any significant difference between the mean ages at death of the non-tumour MCN $F_1$ mice and the non-tumour MNC $F_1$ females.

From this analysis it appears that the higher incidence (by 24·5 per cent) of local tumours in the females of the MNC $F_1$ cross is real and not due to any difference in length of life. While nothing definite can be stated about the MNC $F_1$ males, it is believed that had the non-tumour males survived longer, a corresponding difference of susceptibility would have shown itself.

As, of the parent strains, the CBA mice were the more susceptible the difference between the $F_1$ reciprocal hybrids cannot be maternal in origin and at this time no explanation of the phenomenon can be offered.

4. *Histology of local tumours.*

As the investigation of local tumours was not the main object of the experiment, no attempt was made to carry out a detailed histological examination of every tumour. In fact, most of the tumours produced in the early months of the work were examined ; these amounted to about 150, mainly in the pure strains and in the first 2 hybrid generations, with occasional ones later. The tumours seen were of the various types described by Bonser and Orr (1939) ; the greatest number were subcutaneous fibrosarcomata, of varying degrees of fibrosis ; there were a few pure fibromata, and many spindle and polymorphous-celled sarcomata. Giant cells were frequently present. Where the tumours infiltrated muscle, the result at times resembled a rhabdomyosarcoma. Keratinising epitheliomata were seen, also several mammary carcinomata in some of which sarcoma cells were also present ; lipomata and fibrolipomata also occurred.

5. *Recurrence of local tumours after removal.*

When it became obvious that no progress was being made towards the establishment of lines resistant to the local action of the carcinogen, an attempt was made to prolong the lives of hybrid mice which developed local tumours, by removing a number of these as soon as they were discovered.

Tumours were removed from, or treated with micro-wave radiation (England, 1950) in, 80 MCN and 153 MNC mice at ages varying from 3 to 20 months. Survival times varied from a few days to 16·5 months, and in more than half the cases tumours recurred in times varying from 14 days to 13·5 months after the operation. In 35 cases tumour recurrences were removed or treated a second or even a third time, but in only half of these was there no further recurrence. Post-mortem findings included a large variety of neoplasms such as lung adenoma, mammary carcinoma, non-glandular stomach papilloma, hepatoma, sarcoma of uterus and of bone, stomach epithelioma, etc., many of which develop in older animals and would not have come to light had the local tumours not been removed. In spite of the high percentage of recurrences after the operation therefore the procedure had proved to be of some value.

SUMMARY.

1. Two inbred strains of mice, CBA and NBT, were crossed reciprocally. The hybrids were inbred for 12 generations. Half of each $F_1$ litter received one subcutaneous injection of methylcholanthrene and the descendants of the injected mice for another 9 generations received similar treatment.

2. The local reaction of the inbred strains and of their reciprocal hybrids to the carcinogen is reported.

3. The CBA mice were much more susceptible to the development of local tumours than were the NBT mice. Tumours appeared at the site of injection in 76 per cent of 100 CBA mice and in 39·7 per cent of 131 NBT mice. There was no difference between the strains as regards the age at death of tumour mice.

4. The 10 generations of NBT/CBA hybrids were more susceptible than the 10 generations of reciprocal CBA/NBT mice; 73·4 per cent of 669 NBT/CBA females and 66·4 per cent of 770 NBT/CBA males and 62·8 per cent of 538 CBA/NBT females and 59·6 per cent of 542 CBA/NBT males developed local tumours. There was no difference between the ages at death of tumour mice of the two hybrid strains.

This work was carried out under a research grant from the North of England Council of the British Empire Cancer Campaign. Acknowledgement is gratefully made to Mr. H. Campbell for advice on the statistical analysis of the results.

REFERENCES.

Bonser, G. M., and Orr, J. W.—(1939) *J. Path. Bact.*, **49**, 171.
Boyland, E., and Warren, F. L.—(1937) *Ibid.*, **45**, 171.
Burdette, W. J., and Strong, L. C.—(1943) *Cancer Res.*, **3**, 13.
England, T. S.—(1950) *Ann. Rep. Brit. Emp. Cancer Campgn*, **28**, 180.
Miller, E. W., and Pybus, F. C.—(1942) *J. Path. Bact.*, **54**, 155.—(1945) *Cancer Res.*, **5**, 84.
Pybus, F. C., and Miller, E. W.—(1938) *Amer. J. Cancer*, **33**, 98.—(1940*a*) *Ibid.*, **40**, 47.—(1940*b*) *Ibid.*, **40**, 54.
Strong, L. C.—(1940) *Ibid.*, **39**, 347.—(1945) *J. nat. Cancer Inst.*, **5**, 339.—(1950) *Brit. J. Cancer*, **4**, 315.